# Localization and Characterization of STRO-1^+^ Cells in the Deer Pedicle and Regenerating Antler

**DOI:** 10.1371/journal.pone.0002064

**Published:** 2008-04-30

**Authors:** Hans J. Rolf, Uwe Kierdorf, Horst Kierdorf, Jutta Schulz, Natascha Seymour, Henning Schliephake, Joanna Napp, Sabine Niebert, Helmuth Wölfel, K. Günter Wiese

**Affiliations:** 1 University Hospital, Department of Maxillofacial Surgery, Clinical Research, University of Goettingen, Goettingen, Germany; 2 Department of Biology, University of Hildesheim, Hildesheim, Germany; 3 Wildlife Biology and Game Management, Faculty of Forest Sciences and Forest Ecology, University of Goettingen, Goettingen, Germany; Baylor College of Medicine, United States of America

## Abstract

The annual regeneration of deer antlers is a unique developmental event in mammals, which as a rule possess only a very limited capacity to regenerate lost appendages. Studying antler regeneration can therefore provide a deeper insight into the mechanisms that prevent limb regeneration in humans and other mammals, and, with regard to medical treatments, may possibly even show ways how to overcome these limitations. Traditionally, antler regeneration has been characterized as a process involving the formation of a blastema from de-differentiated cells. More recently it has, however, been hypothesized that antler regeneration is a stem cell-based process. Thus far, direct evidence for the presence of stem cells in primary or regenerating antlers was lacking. Here we demonstrate the presence of cells positive for the mesenchymal stem cell marker STRO-1 in the chondrogenic growth zone and the perivascular tissue of the cartilaginous zone in primary and regenerating antlers as well as in the pedicle of fallow deer (*Dama dama*). In addition, cells positive for the stem cell/progenitor cell markers STRO-1, CD133 and CD271 (LNGFR) were isolated from the growth zones of regenerating fallow deer antlers as well as the pedicle periosteum and cultivated for extended periods of time. We found evidence that STRO-1^+^ cells isolated from the different locations are able to differentiate *in vitro* along the osteogenic and adipogenic lineages. Our results support the view that the annual process of antler regeneration might depend on the periodic activation of mesenchymal progenitor cells located in the pedicle periosteum. The findings of the present study indicate that not only limited tissue regeneration, but also extensive appendage regeneration in a postnatal mammal can occur as a stem cell-based process.

## Introduction

The annual regrowth of deer antlers is the only example of regeneration of a complete, anatomically complex appendage in a mammal, and antlers are therefore of high interest to regeneration biologists [1]–[6]. Antlers are cast and regenerated from permanent bony protuberances of the frontal bones, called pedicles. After antler casting, the bone wound on the top of the pedicle is bordered by the pedicle periosteum and the pedicle skin [7], [8]. Wound healing and epithelialization as well as formation of an antler bud occur very rapidly and, in larger species like the red deer (*Cervus elaphus*), the new antler elongates at an average rate of about 1 cm per day [3]. In contrast to mammals, lower vertebrates have a striking capacity to regenerate complex structures. The epimorphic regeneration involves progenitor cells created through reprogramming of differentiated cells or through the activation of resident stem cells [9], [10]. Exploring the mechanisms of antler regeneration may provide crucial insights to better understand why mammals are unable to regenerate amputated limbs and, with regard to medical treatments, might even provide information that helps to overcome this inability some day. Richard J. Goss, one of the most prominent researchers in the field of antler regeneration during the second half of the 20^th^ century, recognized these chances very clearly and must be credited for linking the study of antler regeneration to regeneration biology in general [2], [3], [11], [12].

The source of the cells that give rise to the regenerating antler has been a matter of controversy. Wislocki [13] and Goss [12], [14] suggested that these cells originate from the pedicle dermis. Currently, however, most workers in the field consider the periosteum of the pedicle to be the source of the cells forming the regenerating antler [7], [8], [15]–[20]. The pedicle periosteum is a derivative of the antlerogenic periosteum that builds up the pedicle and the first antler [21]–[23]. Recently, it has been hypothesized that antler regeneration is a stem cell based process [5], [7], [8], [15], [23]. According to this view, stem cells located in the pedicle periosteum give rise to progenitor cells of different lineages, such as chondro- and osteoprogenitors [7]. However, thus far direct evidence for the existence of stem cells in the pedicle periosteum and the growing the antler was lacking. As part of an ongoing research project, we searched for the presence of cells positive for known stem cell markers in pedicles and growing antlers of fallow deer (*Dama dama*) [24], [25]. In addition, we isolated and cultivated stem cells derived from the deer antler/pedicle and investigated their proliferation and differentiation properties.

## Results

### Immunohistochemical localization of STRO-1^+^ cells in the antler and pedicle

**Figure 1:**
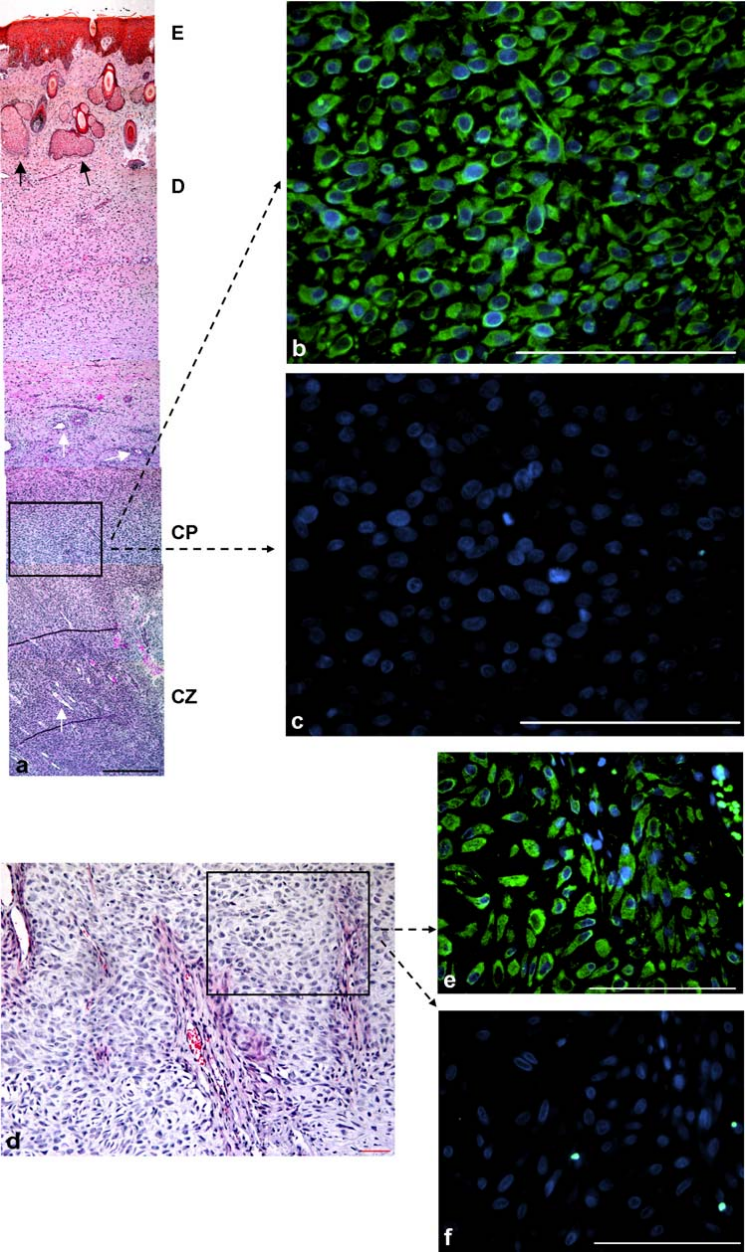
STRO-1^+^ cells in the cambial layer of the perichondrium and the cartilaginous zone of an antler. Paraffin embedded biopsy samples of a velvet antler from a 4 yr-old fallow buck (*Dama dama*); samples were taken 46 days after onset of regeneration. (a) Cross section of brow tine about 1 cm below the tip, overview, (E) epidermis, (D) dermis, (CP) cambial layer of the perichondrium, (CZ) cartilaginous zone, white arrows = vessels, black arrows = sebaceous glands; HE-staining, scale bar: 500 µm. (b) STRO-1^+^ cells in the cambial layer of the perichondrium [STRO-1 antibody combined with an anti-mouse IgM secondary antibody conjugated with fluorescence dye (FITC), nuclei counter-stained with Hoechst 33342], scale bar: 100 µm. (c) Negative control, cambial layer of the perichondrium, same staining as (b) without STRO-1 antibody, scale bar: 100 µm, identical exposure times for pictures (b) and (c). (d) Cross section of part of a main beam, cartilaginous zone, HE-staining, scale bar: 100 µm. (e) STRO-1^+^ cells within the cartilaginous zone [same staining as (b)], scale bar: 100 µm. (f) Negative control, comparable area of the cartilaginous zone, same staining as (e) without STRO-1 antibody, scale bar: 100 µm, identical exposure times for pictures (e) and (f).

In the regenerating antler, a high density of STRO-1^+^ cells was found in the cambial layer of the perichondrium (Fig. 1a–c) and within the chondrogenic growth zone (Fig. 1d–f) present at the tips of the main beam and the antler tines.

**Figure 2:**
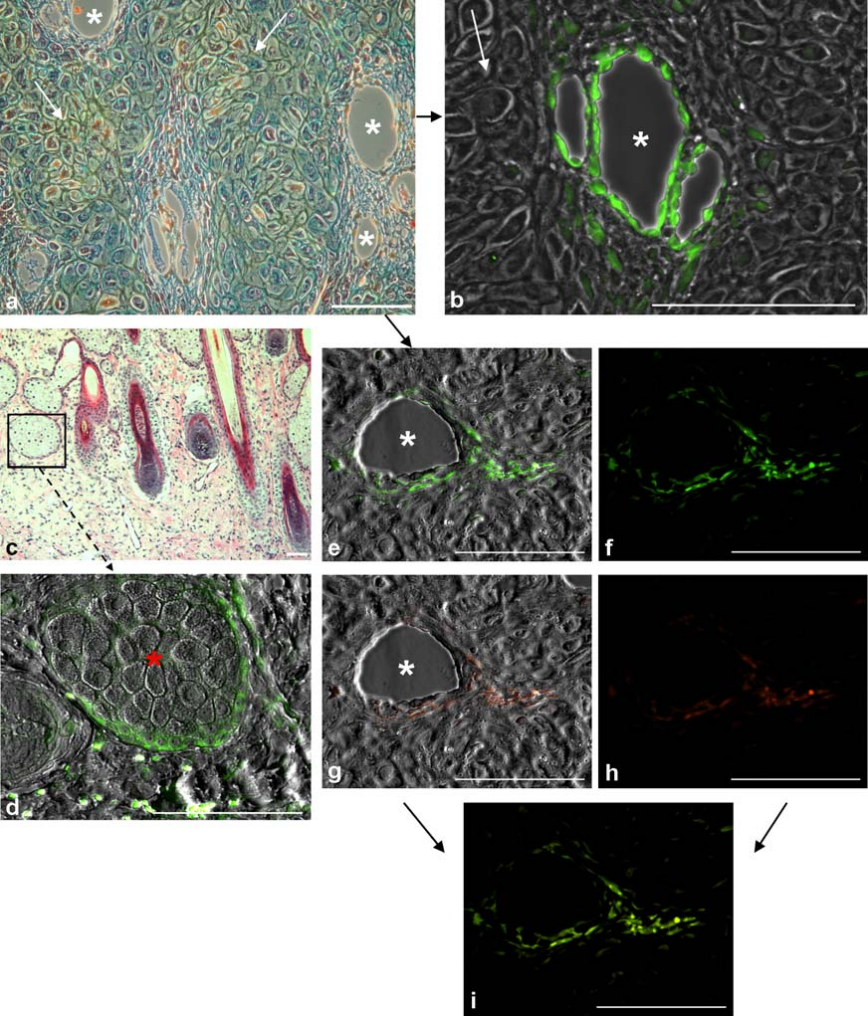
STRO-1^+^ cells in different locations of a velvet antler. (a-i) Paraffin embedded biopsy samples of velvet antler (main beam, cross-sections, samples taken about 1 cm below the tip), 9 yr-old fallow buck (*Dama dama*); samples were taken 74 days after onset of regeneration; scale bars: 100 µm. (a) Part of the cartilaginous zone, numerous blood vessels are located in the area between the cartilaginous trabeculae, white asterisks = vessels, white arrows = chondrogenic cells, Movat-staining. (b) Perivascular and endothelial cells staining positive for the STRO-1 antibody (fluorescence dye = FITC), phase-contrast picture. (c) Part of velvet skin containing hair follicles and sebaceous glands (black square), HE-staining. (d) STRO-1^+^ cells at the base of a sebaceous gland, red asterisk = sebaceous gland, varel-contrast picture. (e–i) Perivascular cells in the cartilaginous zone. (e) STRO-1^+^ cells, white asterisk = vessel, varel-contrast picture. (f) Same picture as (e), STRO-1^+^ fluorescence only. (g) CD271^+^ cells [CD271 antibody combined with an anti-mouse IgG secondary antibody conjugated with fluorescence dye (Alexa Fluor 546)], white asterisk = vessel, varel contrast picture. (h) Same picture as (g), CD271^+^ fluorescence only. (i) Merged image of (f) and (h).

In the more proximally located cartilaginous zone, STRO-1^+^cells were detected in the perivascular tissue and in the vascular endothelium (Fig. 2a, b). Furthermore, STRO-1^+^ cells were detected at the base of sebaceous glands within the velvet (Fig. 2c, d). In some perivascular areas, STRO-1^+^ cells (Fig. 2e, f) were found to be also slight positive for the CD271 marker (Fig. 2g, h), as shown in the merged image (Fig. 2i)

**Figure 3:**
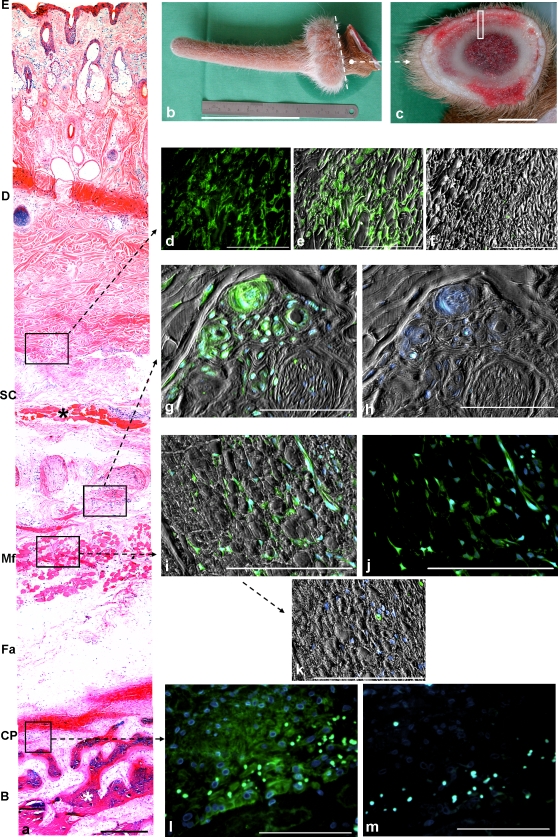
STRO-1^+^ cells in different areas of the pedicle. (a) Methylmetacrylate (Technovit® 9100 New) embedded sample of the pedicle shown in (b) and (c); cross-section, overview, HE-staining. (E) epidermis, (D) dermis, (SC) subcutaneous tissue with superficial muscle (asterisk), (Mf) Part of the frontoscutular muscle, (Fa) fascia (*tissue slightly lacerated during histological processing*) , (CP) cambial layer of the periosteum, (B) pedicle bone; white asterisk = bony trabeculae, scale bar: 500 µm. (b) Left pedicle and primary velvet antler of a 1 yr-old fallow buck (*Dama dama*), the antler was cut below the coronet (dashed line) to obtain a cross-section of the distal pedicle, scale bar: 10 cm. (c) Cross-section of the distal pedicle shown in (b); white rectangle marks the area shown in (a); scale bar:1 cm. For all pictures (d-m): [STRO-1 antibody was combined with an anti-mouse IgM secondary antibody conjugated with fluorescence dye (FITC), nuclei were counter-stained with Hoechst 33342]. (d,e) STRO-1^+^ cells within the reticular layer of the dermis, located between thick collagen fibres; (d) STRO-1^+^ fluorescence only, same area as (e); (e) Fluorescence combined with varel-contrast picture; (f) Negative control; similar area as shown in (e); the small green dots are erythrocytes marked by the fluorescence dyes; identical exposure times for pictures (e) and (f), scale bars: 100 µm. (g) Vascular associated STRO-1^+^ cells within the subcutaneous tissue, varel-contrast picture, scale bar: 100 µm. (h) Negative control; same area as shown in (g); identical exposure times for pictures (g) and (h), varel-contrast picture, scale bar: 100 µm. (i–k) STRO-1^+^ cells between fibres of the frontoscutular muscle, scale bars: 100 µm; (i) Fluorescence combined with varel-contrast picture; (j) STRO-1^+^ fluorescence only, same area as (i); (k) Negative control, similar area as shown in (i); varel-contrast picture, identical exposure times for pictures (i) and (k); the bright green dots in picture (k) are erythrocytes marked by the fluorescence dyes. (l) STRO-1^+^ cells within the cambial layer of the periosteum; scale bar: 100 µm. (m) Negative control, similar area as shown in (l); scale bar: 100 µm, identical exposure times for pictures (l) and (m); the bright dots in pictures (l) and (m) are erythrocytes marked by the fluorescence dyes.

STRO-1^+^ cells were also present in different tissues of the pedicle of the yearling fallow buck (Fig. 3a–c). Located between thick collagen fibres, STRO-1^+^ cells were detected within the reticular layer of the dermis (Fig. 3a, d–f). In addition, STRO-1^+^ perivascular cells were located in the subcutaneous tissue (Fig. 3a, g, h). Corresponding to the situation in the regenerating antler, a high density of STRO-1^+^ cells was found in the cambial layer of the pedicle periosteum (Fig. 3a, l, m). Moreover, groups of STRO-1^+^ cells resembling satellite cells were observed between muscle fibers of the frontoscutular muscle (Fig. 3a, i–k).

### Cell sorting

**Figure 4:**
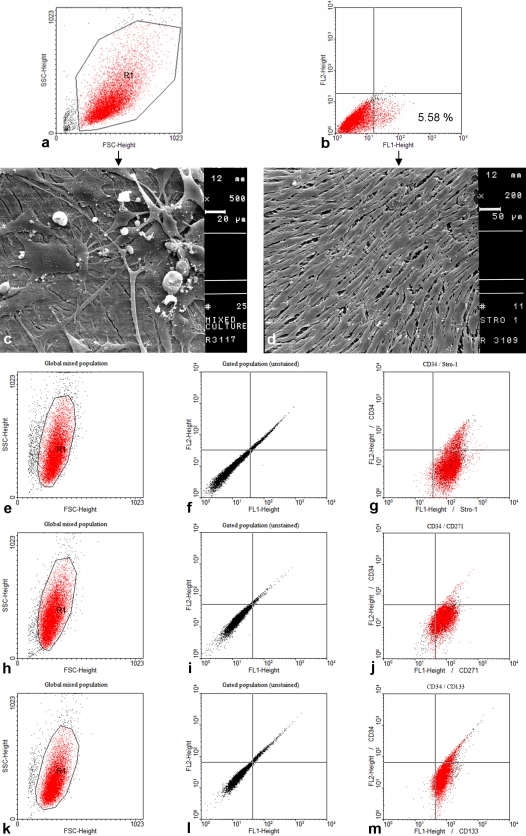
Isolation of STRO-1^+^, CD271^+^ and CD133^+^ cells derived from regenerating deer antler and pedicle periosteum. The mixed cell populations were analysed by flow cytometry (FACS). (a,b) Mixed population of cells derived from the antler growth zone (b) Percentage of STRO-1^+^ cells within the gated population (R1). (c) Scanning electron microscopy (SEM) picture of a mixed antler cell population, scale bar: 20 µm (×500). (d) SEM picture of a pure STRO-1^+^ cell population, scale bar: 50 µm (×200). Samples shown at pictures (c) and (d) were prepared after cell cultures had reached confluence. (e–m) Mixed cell population derived from the pedicle periosteum; (e,h,k) Global mixed populations (FSC/SSC); (f,i,l) Gated populations (unstained), cells of gate R1 (FSC/SSC) plotted as FL2 as a function of FL1; (g) Double staining (CD34/STRO-1), FL1 = STRO-1, FL2 = CD34; (j) Double staining (CD34/CD271), FL1 = CD271, FL2 = CD34; (m) Double staining (CD34/CD133), FL1 = CD133, FL2 = CD34.

Primary mixed cell cultures were established from tissue samples of the chondrogenic growth zones of primary and regenerating antlers as well as the pedicle periosteum. To obtain pure cultures of STRO-1^+^, CD271^+^ and CD133^+^ cells, we used these markers for cell sorting. Applying fluorescence-activated cell sorting (FACS), we obtained 3.5%–5.5% STRO-1^+^ cells from the primary mixed cell cultures (Fig. 4a, b). Scanning electron microscopy (SEM) showed that, in contrast to mixed antler cell cultures, the sorted STRO-1^+^ cells grow as a homogeneous cell population (Fig. 4c, d). CD271^+^ and CD133^+^ cells can be isolated at high purity and grown with homogenous morphology as well (data not shown).

Primary cultures of the pedicle periosteum were analyzed by FACS and STRO-1^+^ (Fig. 4e–g), CD271^+^ (Fig. 4h–j) and CD133^+^ (Fig. 4k–m) cells were found to be CD34 negative.

Magnetic cell sorting (MACS®) gave up to 13.4% STRO-1^+^ cells in mixed cultures derived from the chondrogenic growth zone of antlers of adult fallow deer (Table 1). Highest numbers of STRO-1^+^ cells (17.3%) were detected in primary cultures derived from the pedicle periosteum of the yearling fallow buck (Table 1). Cells positive for CD14 -, CD34 -, CD105 -, CD133 (human)-and CD271 (LNGFR)–surface markers were present in mixed cultures derived from regenerating antlers (Table 1).

**Table 1:** MACS-Analyses of “mixed” cell cultures derived from regenerating antlers.

Antibody	Positive cells [%]
CD34	11.8 *
CD105	4.6 *
CD14	1.7 *
CD271 (LNGFR)	2.8–4.8 **
CD133 (human)	14.3–16.5 **
STRO-1	17.3 (pedicle periosteum of a yearling fallow buck/primary culture)
STRO-1	3.5–13.4 ** (antler growth zone/adult *fallow deer* )

Percentages of cells positive for different surface markers

* Single analysis/Second passage of cells derived from the antler growth zone of an adult fallow deer.

** Values obtained from different culture analyses (analysed were primary cultures till third passages)

### RT-PCR analyses

**Figure 5:**
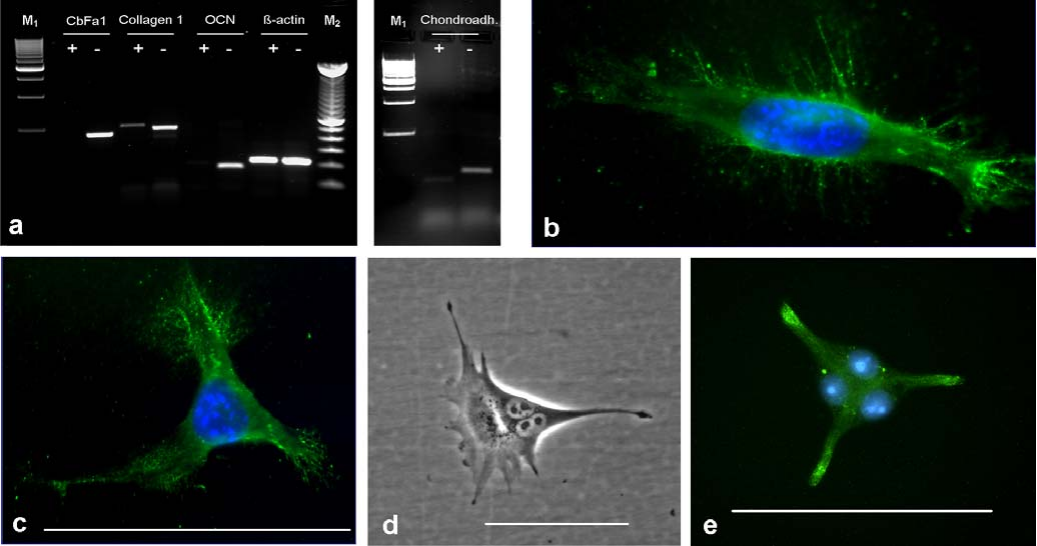
Expression profiles and morphology of isolated STRO-1^+^ cells. (a) Expression profiles of STRO-1 negative versus STRO-1^+^ cells. RT-PCR was used to detect the mRNA of specific markers for the osteogenic [Collagen 1, cbfa 1, osteocalcin (OCN)] and the chondrogenic lineages (chondroadherin). Expression of deer ß-actin was used for standardization. (+) = STRO-1^+^ cells, (−) = STRO-1 negative cells, (M_1_) = Marker: 500 bp DNA ladder, (M_2_) = Marker: 100 bp DNA ladder. (b,c) Typical morphology of STRO-1^+^ cells isolated from fallow deer antler cell cultures [STRO-1 antibody combined with fluorescence dye (FITC), nuclei counter-stained with Hoechst 33342], scale bar: 100 µm. (d,e) STRO-1^+^ stem cells with three nuclei, (d) phase contrast picture; (e) same staining as shown in (b) and (c); scale bars: 100 µm.

RT-PCR analyses showed that under standard culture conditions [DMEM (Gibco) + 10% fetal calf serum], the cultured STRO-1^+^ cells did not express key markers of the osteogenic (cbfa 1, osteocalcin) or chondrogenic (chondroadherin) lineages (Fig. 5a). However, a weak expression of collagen 1 was noted, suggesting that a few cells were already differentiated. In contrast, the STRO-1 negative antler cell populations showed marked expressions of the above key markers indicating the presence of cells of the osteogenic and chondrogenic lineages. Sequencing of PCR products amplified using collagen 1 primers revealed 100% identity with published sequences of human, bovine and mouse collagen 1. In addition, PCR products amplified using GAPDH and ß-actin primers showed also 100% identities with published sequences of roe deer, human, bovine, mouse GAPDH and red deer, human, bovine, mouse ß-actin, respectively.

### *In vitro*–morphology, proliferation and differentiation capacity of STRO-1^+^ cells

Examples of the morphology of non-confluent STRO-1^+^ cells are shown in Fig. 5b–e. Occasionally, we also observed “atypical” cells with three nuclei (Fig. 5d, e), a phenomenon that to our knowledge has not previously been reported for stem cells in culture.

**Figure 6:**
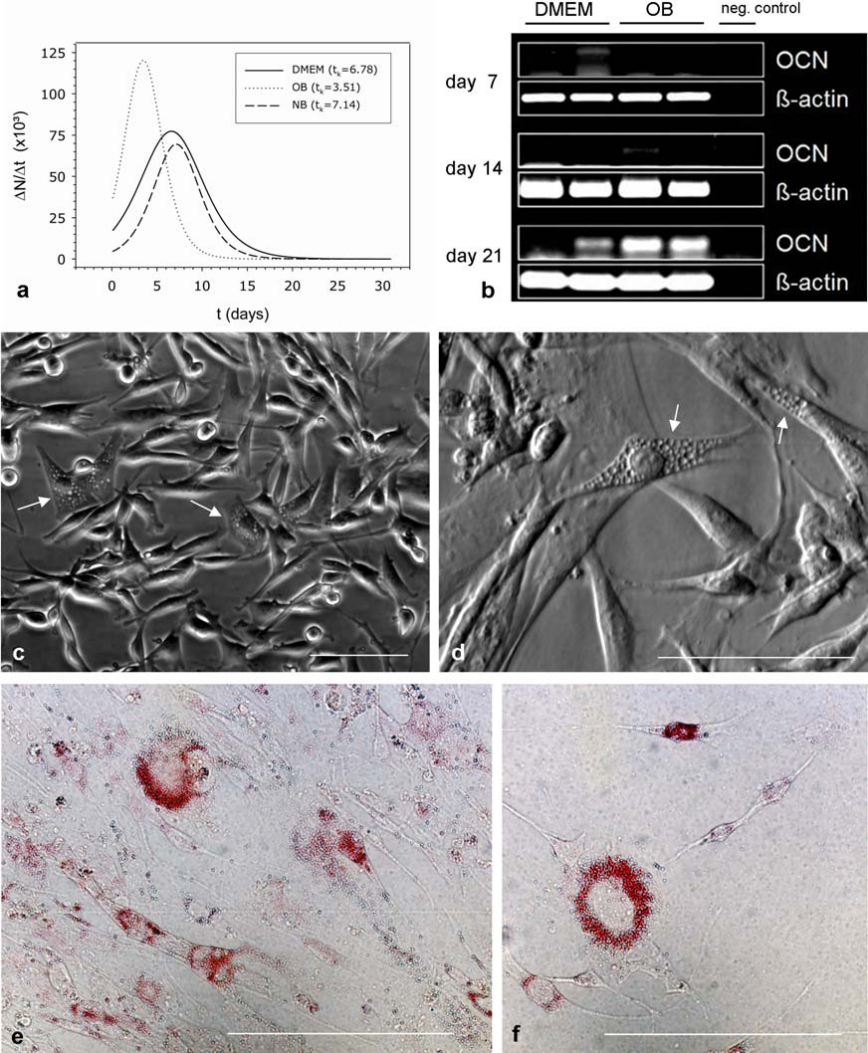
Growth and differentiation of STRO-1^+^ cells in different culture media. (a) Time –dependent increase in cell numbers (ΔN/Δt) in Dulbecco's Minimal Eagle Medium (DMEM), osteoblast proliferation medium (OB), and NeuroBasal medium containing 50 ng/ml nerve growth factor (NB). The peak values of the curves coincide with the time when the cells reached confluence (t_k_), culture well area = 2 cm^2^. (b) Expression of osteocalcin in isolated STRO-1^+^ cells cultured for several weeks in DMEM and OB-medium. RT-PCR was used to detect the mRNA of osteocalcin (OCN); expression was investigated at culture days 7, 14 and 21. (c,d) STRO-1^+^ cells after four days of induced adipogenic differentiation in adipocyte differentiation medium starting with intracellular lipid formation (white arrows), (c) phase contrast, (d) varel contrast; scale bars: 100 µm. (e,f) STRO-1^+^ cells after 10 days culture in adipocyte differentiation medium. Cells were fixed, stained for lipid accumulation (Oil Red O) and observed under a light microscope; scale bars: 100 µm.

To investigate the influence of different culture media on the proliferation of STRO-1^+^ cells, we cultivated cells for one month in Dulbecco's minimal eagle medium [DMEM (Gibco) + 10% FCS], osteoblast growth medium (OB) + supplement mix (both Promocell) or NeuroBasal medium (NB/Gibco) containing 50 ng/ml nerve growth factor (NGF 7S/Invitrogen). Proliferation rates of STRO-1^+^ cells differed between these media (Fig. 6a). Proliferation rates per day (ΔN/Δt) were highest in osteoblast medium. The peaks of the different growth curves coincide with cell confluence in culture wells. In OB-medium confluence was reached after 3.51 days in culture whereas in DMEM and NB–medium confluence occurred after 6.78 and 7.14 days, respectively. Afterwards, proliferation decreased dramatically in all media to minimal values within 5-6 days. A distinct osteocalcin expression could be observed in osteoblast growth medium at day 21, indicating a differentiation of STRO-1^+^ cells into osteoblasts (Fig. 6b).

After four days of induced fat differentiation in NH AdipoDiff Medium, STRO-1^+^ cells started with intracellular lipid formation (Fig. 6c, d). Upon prolonged culture in adipocyte medium for 10 days, many cells had accumulated cytoplasmic lipid droplets that stained positive with Oil Red O (Fig. 6e, f).

## Discussion

The most important finding of the present study is the demonstration of STRO-1^+^ stem cells in different locations of the primary and regenerating antler as well as in the pedicle of fallow deer. FACS analyses revealed that primary cell cultures derived from the pedicle periosteum contain STRO-1^+^, CD271^+^ and CD133^+^ cell populations that are negative for CD34 (marker for hematopoietic progenitors). Therefore, these cells can be defined as mesenchymal progenitor cells. These findings strongly support the view that the annual antler regeneration represents a stem cell-based process. The results are consistent with the hypothesis that the regenerating antler is build up by the progeny of mesenchymal stem cells located in the cambial layer of the pedicle periosteum [5], [7], [8], 15, 23. It has recently been shown that stem cell populations exist in “niches”—specific anatomical locations that regulate how the stem cells participate in tissue generation, maintenance and repair [26], [27]. We assume that such a “stem cell niche” is located in the cambial layer of the periosteum and that the regeneration of antlers is dependent on the periodic activation of these stem cells. In the pedicle, STRO-1^+^ cells resembling satellite cells [28], [29] were also found adjacent to muscle fibres (frontoscutular muscle). The existence of possible satellite cells in this area remains to be elucidated by further studies. In addition, it is also necessary to find explanations for the presence of STRO-1^+^ cells detected within the reticular layer of the dermis in the pedicle of the juvenile fallow buck. It is conceivable that dermal and epidermal stem cells/progenitor cells are involved in the rapid proliferation of velvet components during antler regeneration [30].

In the regenerating antler, STRO-1^+^ cells were found in a perivascular and vascular endothelial location both in the subcutaneous tissue of the pedicle and in the cartilaginous zone. Immunocytochemistry revealed cells positive for the stem cell marker CD271 at similar locations in the regenerating antler. CD271, also known as low-affinity nerve growth factor receptor (LNGFR), is a marker for the isolation of mesenchymal stem cells directly from bone marrow aspirate [31], [32]. To date, the function of LNGFR on mesenchymal progenitor cells is not clear, but it is discussed that it may have a morphogenic role in the development of the human bone marrow cavity and other organs [31]. Our double-stainings for STRO-1 and CD271 indicate that cells in these perivascular locations within deer antler tissue can be positive for both markers. The meaning of the presence of CD271/LNGFR on STRO-1^+^ cells requires further investigations. Recently, it was reported that the growing tip of the deer antler contains proliferating perivascular cells and possible angioblastic precursors [33]. In addition, in other animal models perivascular cells were described that seemed to be a novel stem cell-like population with the capacity to differentiate into multiple mesenchymal lineages [34]. Further studies are necessary to clarify whether the STRO-1^+^ perivascular cells in the pedicle and regenerating deer antler and the STRO-1^+^/CD271^+^ cells in the regenerating antler represent angioblastic precursors. For that reasons, is has to be elucidated whether STRO-1^+^ and/or CD271^+^ cells can be found in the vascular endothelium at different locations of the regenerating antler. For example, this seems to be the case in Fig. 2b, where the vascular endothelium contains STRO-1^+^ cells.

STRO-1^+^ cells were also observed within the velvet skin of the regenerating antler. We detected STRO-1^+^ cells associated with sebaceous glands. Presence of pluripotent neural crest derived stem cells in the adult mammalian hair follicle has been reported by several authors [35]–[37]. *De novo* formation of hair follicles is a characteristic of velvet skin [1] and follicle-associated STRO-1^+^ cells may play a crucial role in this process.

Recently, different groups have found clues to the presence of stem cells/progenitor cells in the pedicle periosteum as well as in primary and regenerating antlers [16], [24], [38]–[40]. Here we proved for the first time the existence of STRO-1^+^, CD271^+^ and CD133^+^ cells in different areas of the pedicle and the primary and regenerating fallow deer antler. Preliminary studies on cell cultures derived from the growth zone of regenerating red deer antlers also revealed the presence of STRO-1^+^ cells (1.5–13.3%, 35 analyses) (Kuzmova et al., *unpublished results*).

We were able to sort (FACS and MACS®) and cultivate the STRO-1^+^, CD271^+^ and CD133^+^ cells as “pure” cultures. Expression profiles of isolated STRO-1^+^ cells versus STRO-1**^−^** cell populations suggest that indeed the STRO-1^+^ cells represent a population of progenitor cells. In addition, preliminary MACS® analyses proved the existence of cells positive for the stem cells/progenitor cell markers CD34, CD105, CD133 and CD271 (LNGFR) in the regenerating antler. Since regenerating antler tissue contains also hematopoietic cells and progenitors in great quantities it is not surprising that we were able to detect CD34^+^ cells in primary mixed cultures derived from this tissue. In contrast, primary cultures from pedicle periosteum did not contain CD34^+^ cells.

STRO-1^+^ cells showed different growth patterns and different cell shapes in DMEM, OB–and NB–medium (*data not shown*). Remarkably STRO-1^+^ cells exhibited highest proliferation rates in the first four days in culture in osteoblast differentiation medium compared to DMEM and NB-medium. The reason for this difference in proliferation rates remains to be elucidated. The occasional occurrence of STRO-1^+^ cells with three nuclei (Fig. 5d, e) might be related to the rapidity of the proliferation process.

The present study showed that *in vitro* the differentiation potential of STRO-1^+^ cells is not restricted to the osteogenic and chondrogenic lineages. In addition to their ability to differentiate *in vitro* into cell types that naturally occur in growing antlers (e.g. osteoblasts), under appropriate culture conditions STRO-1^+^ cells were also able to differentiate into adipocytes. In contrast to long bones, adipogenesis does not occur in regenerating antlers. For that reason, we conclude that STRO-1^+^ cells in deer antlers represent a population of at least bi- or tripotent mesenchymal progenitor cells. These results are in accordance with the finding that adipogenesis can be induced in cultured donor cell lines derived from antlerogenic periosteum of male red deer (*Cervus elaphus*) [40].

In conclusion, the present study provides evidence for the contribution of stem cells in the process of antler regeneration. We suggest that the development of primary antlers and the yearly replacement of antlers in adult deer are both stem cell-dependent processes. We further suggest that antler regeneration involves the activation of stem cells located in a niche in the cambial layer of the pedicle periosteum. The presence of stem cells/progenitor cells observed in different locations of primary and regenerating antlers suggests that these cells play a role both for the formation of the interior component (e. g. bone and cartilage) as well as the external component (velvet skin) of the growing antler. The results of the present study also suggest that, in the case of antlers, extensive regeneration of a histological complex appendage in a postnatal mammal is triggered by activation of resident stem cells located in different “niches”, e.g. stem cells located in the pedicle periosteum of the deer. This mode of regeneration is different from that occurring during limb regeneration in urodele amphibians and fin regeneration in teleost fish [29], [41], which involve large-scale dedifferentiation and reprogramming of cells in the amputation stump. Based on the findings on antler regeneration it could thus be speculated that induction of dedifferentiation in the stump tissue may also not be an indispensable step in promoting limb regeneration in mammals. However, there is still much to be learned about the similarities and differences of the mechanisms that prevent the regeneration of amputated limbs in mammals (including deer) and that allow and regulate the periodic regeneration of deer antlers. Antler regeneration may therefore be a useful model for the study of stem cell based regenerative processes in mammals including humans.

## Materials and Methods

### Tissue sampling and cell culture

Tissue samples were obtained from a yearling fallow buck and from five adult fallow bucks, aged between 4 and 9 years. The yearling buck, which was growing its first set of antlers, and one of the five adult bucks were killed and their pedicles and antlers collected. In four of the adult bucks, tissue samples from the antler growth region were obtained with a bioptic punch between day 36 and day 144 after casting of the previous hard antlers. The biopsies were taken 1–2 cm below the growing tip of the regenerating antler. Pedicle and antler tissue samples were used for histology and cell cultures. The latter were established as previously described and did not include pedicle and antler skin [6].

All tissue samples were carried out in compliance with the institutional guidelines on animal husbandry and care/welfare of the University Hospital in Goettingen (*Department for Animal Experiments*) and the Institute for Wildlife Biology and Game Management (*Faculty of Forest Sciences and Forest Ecology*), University of Goettingen, Germany. In addition, the authorization for the experiments was given by the district government Braunschweig, Germany (*permission numbers*: 604.42502/01-21.96 and 604.42502/01-22.96).

### Tissue preparation and histology

Tissue samples were embedded in either paraffin (soft tissue) or methylmetacrylate (Technovit® 9100 new, Heraeus Kulzer GmbH, Germany) (undecalcified mineralized specimens) according to Delling [42]. Histological sections were stained with hematoxylin-eosin (HE) and Movat's stain [43]. For scanning electron microscopy cells were fixed in glutaraldehyde, dehydrated in a graded series of ethanol and critical point dried. Specimens were sputtered with gold-palladium and viewed in a Zeiss DSM 960 scanning electron microscope.

### Flow cytometry and MACS® analyses

When the cultured cells approached confluence, they were trypsinised, labeled with STRO-1- [44] (MAB 1038, R&D Systems, Germany and Developmental Studies Hybridoma Bank, Iowa, USA), CD133-, CD271- (Miltenyi Biotec Inc., Bergisch Gladbach, Germany) and CD34- (Immunotech and Miltenyi Biotec) antibodies, and analyzed by flow cytometry (FACSVantage™ SE/Becton Dickinson). In addition, for positive cell selection using magnetic cell sorting (MACS®, Miltenyi Biotec), mixed cell cultures from the antler growth region and pedicle periosteum were labeled with CD14-, CD34-, CD105-, CD133-, CD271 (LNGFR)–MicroBeads (Miltenyi Biotec) as well as a STRO-1 antibody (MAB 1038, R&D Systems, Germany) coupled with IgM–MicroBeads (Miltenyi Biotec). Cells derived from primary cultures or up to third passages were used for selection.

### Immunocytochemistry

Immunocytochemistry was performed on paraffin embedded as well as methylmetacrylate embedded samples from the pedicle and from primary and regenerating antlers to investigate possible localizations of stem cells within the tissues. The STRO-1 antibody was used in combination with an anti-mouse IgM secondary antibody conjugated with a fluorescent dye [Fluorescein isothiocyanate (FITC), Becton Dickinson]. The CD271 marker (Miltenyi Biotec) was used in combination with an anti-mouse IgG secondary antibody conjugated with Alexa Fluor 546 (Molecular Probes). Negative controls were performed using conjugated secondary antibodies only. The nuclei were counter-stained with Hoechst 33342 fluorescent dye (Invitrogen). For target retrieval of specimens a microprocessor controlled Pascal pressure chamber (DakoCytomation) was used (30 seconds at 123 °C).

### RT-PCR

Total cellular RNA was isolated, after MACS separation, from both STRO-1^+^ and STRO-1^−^ cells using the RNeasy Mini Kit (Qiagen, Germany). Reverse transcription was performed with 0.5 or 1 mg of total RNA using the iScript™ cDNA Synthesis Kit (Biorad, Germany). In total, 50 ng of cDNA were used for reverse transcription-polymerase chain reaction (RT-PCR). PCR amplification was carried out using primers for cbfa1, osteocalcin, chondroadherin, type I collagen and deer ß-actin [45] (as house keeping gene). The primer sequences used in this study are summarized in Table 2. Each PCR protocol started with an initial denaturation step of 95°C for 2 min and ended with an additional single step for 7 min at 72°C. PCR-amplifications were carried out as follows: Cbfa1: 30 Cycles at 95°C for 45 sec, annealing at 58°C for 45 sec, and extension for 45 sec at 72°C. Collagen1: 28 cycles at 95°C for 45 sec, annealing at 53°C for 45 sec, and extension for 45 sec at 72°C. Osteocalcin: 32 cycles at 95°C for 45 sec, annealing at 53°C for 45 sec, and extension for 45 sec at 72°C. Deer ß-actin: 23 cycles at 95°C for 45 sec, annealing at 60°C for 45 sec, and extension for 45 sec at 72°C. Chondroadherin: 30 cycles at 95°C for 45 sec, annealing at 55°C for 45 sec, and extension for 45 sec at 72°C. PCR products were analyzed by agarose gel electrophoresis and detected by ethidium bromide staining under UV light. The specificity of the PCR products obtained with Collagen 1-, GAPDH- and deer ß-actin- primers was proven by sequencing (SEQLAB, Göttingen).

**Table 2:** Primer sequences used in RT–PCR analyses

Gene	Nucleotide Sequence (5′-3′)
Cbfa1	for: GTGAGGGATGAAATGCTTGGGAAC
	rev: CATAACCGTCTTCACAAATCCTCCC
Collagen1	for: GACCTCCGGCTCCTGCTCCTCTTAG
	rev: GGACCCATGGGGCCAGGCACGGAAA
Osteocalcin	for: GCCCTCACACTCCTCGCCCTATTGG
	rev: GTCTCTTCACTACCTCGCTGCCCTC
Deer ß-actin	for: CCCAAGGCCAACCGTGAGAAGATG
	rev: GTCCCGGCCAGCCAAGTCCAG
Chondroadherin	for: ACCTGGACCACAACAAGGTC
	rev: CTTCTCCAGGTTGGTGTTGTCC

### Osteogenic and adipogenic differentiation

It has been previously shown that STRO-1^+^ bone marrow cells can differentiate along multiple mesenchymal lineages including adipocytes, osteoblasts and chondrocytes [46], [47]. Differentiation capability of MACS® sorted STRO-1^+^ cells from the antler growth region into cells of the adipocyte lineage was tested using an adipogenic differentiation medium (MACS® NH AdipoDiff Medium, Miltenyi Biotec). STRO-1^+^ cells were first incubated for 3 days in MACS® NH Expansion Medium (Miltenyi Biotec), followed by incubation in AdipoDiff medium for 10 days. Osteogenic and chondrogenic differentiation capabilities of STRO-1^+^ cells from the antler growth region were tested by incubating them immediately after sorting for 3–4 weeks in osteoblast growth medium (PromoCell) + supplement mix (C-27001, PromoCell) and in osteoblast and chondroblast differentiation media from the hMSC functional identification kit (SC006, R&D Systems). In addition, STRO-1^+^ cells were also grown in NeuroBasal medium (Gibco) containing 50 ng/ml nerve growth factor (NGF 7S, Invitrogen). In each experiment, a STRO-1 negative fraction was treated accordingly.

### Oil Red O staining

After incubation in AdipoDiff medium for 4 or 10 days, cells were fixed in 4% paraformaldehyde for 15 min at room temperature, washed three times in PBS, stained for 60 min in a freshly filtered solution of six parts saturated oil red O (Sigma, 0.5 g in 100 ml isopropanol) and four parts ddH_2_O, washed thoroughly in ddH_2_O and finally mounted with DAKO mounting medium.

### Statistical analysis

Cell numbers in the cultures (3≤n≤6) were determined daily by using an electronic cell counter system (CASY®, Schaerfe System, Reutlingen, Germany), starting with the day of seeding. Mean cell numbers were calculated and plotted against time, and regression curves were fitted to the data using Sigma Plot® (Erkrath, Germany). The daily increase in cell number (1. derivation of the regression curve) was then calculated based on the regression.
